# Historical Sketch of Some of the London Hospitals

**Published:** 1904-02-06

**Authors:** J. E. R. Stephens


### HISTORICAL SKETCH OF SOME OF THE LONDON HOSPITALS.—I.

By J. E. R. STEPHENS.

THE word hospital is derived from the Latin *hospitalis*, and this again from the noun *hospes*, a host or guest. Hospital in law is used in England to denote an eleemosynary corporation founded for the purpose of supporting certain descriptions of persons; whereas in Scotland it more frequently signifies an endowment for the education as well as support of children. In both countries it is also used, popularly to denote an institution for dispensing medical assistance to the poor gratuitously.

Although in ancient times there may have been places for the reception of strangers and travellers, it seems at least doubtful if there was anything of the nature of a charitable institution for the reception of the sick such as existed after the introduction of Christianity. Among the Greeks there seems little evidence of the existence of establishments for the sick. Even for sick and wounded Roman soldiers but little provision seems to have been made, although we do not know much of the *valetudinarium*, which appears to have existed in a Roman camp. That the Romans had a medical staff has been shown by the monuments discovered in Great Britain and the subject has been carefully examined by the late Sir James Simpson (Transactions of the Society of Antiquaries of Scotland, etc.). Amongst the earliest hospitals on record is that said to have been founded by Valens in Caesarea 370-380 A.D. and the one built at Rome by Fabiola, a Roman lady and friend of St. Jerome, although like most others of that and even later times both were probably almshouses as well.

The origin of our present hospitals must, however, be looked for in the monastic arrangements for the care of the sick and indigent. Every monastery had its *infirmaria*, managed by an *infirmarius*, in which not only were sick and convalescents treated, but also the aged, the blind, the weak, etc., were housed. In course of time separate buildings were erected for the purpose, and special revenues augmented from time to time by benefactions, appropriated for their maintenance. In numerous instances, however, the hospitals were converted into benefices by the priests, and the scandal had to be dealt with by the authority of general councils, which, like that of Vienna, forbade the practice. About the earliest distinct record of the building of a hospital in England is in the life of Lanfranc, Archbishop of Canterbury, who in 1080 founded two, one for leprosy and one for ordinary diseases. The former is referred to in the "Vie de St. Thomas le Martyr," a work of the twelfth century. The establishments for the sick remained in the hands of the clergy until the Reformation, when some of the monasteries and church property were appropriated and set apart for the use of the sick. Of these the most noted instances were St. Bartholomew's in Smithfield, St. Thomas's in the Borough, Bethlehem or Bedlam, Bridewell, and Christ's Hospital, which were long known as the "Five Royal Hospitals." St. Bartholomew's was a priory, founded in 1102. It was handed over to the citizens of London as a hospital in 1547; it escaped the fire of 1666, and was rebuilt in 1729. St. Thomas's was founded by Richard, prior of Bermondsey, in 1213, surrendered in 1538, and purchased by the mayor and citizens of London in 1551, and opened for 260 sick. It was incorporated in 1553, rebuilt in 1693, added to in 1732, removed temporarily to Surrey Gardens in 1862, and finally transferred to Lambeth, its present site, in 1871. Bethlehem (or Bedlam) was a priory, founded by Simon Fitzmary in 1247. In 1547 it was handed over by Henry VIII. for the reception of lunatics. It was rebuilt in 1676, and wings were added in 1733. The present building was constructed in 1810.

The mediæval hospitals were generally established for the reception and relief of lepers, whose malady was one of the scourges of Europe. These leper hospitals were very commonly in England and in Scotland called "Spitals"; hence the frequency of such names of places as Spital, Spitalfields, etc. The leper hospitals, and other kinds of the old hospitals, disappeared with the improvement of society, and substitutes for them on a broader scale began to be established in the modern form of hospitals. Hospitals for the sick and wounded are sometimes called infirmaries. Under whatever designations institutions of this kind are now established in all parts of the civilised world, supported as in England, on a principle of charity, or, as in France chiefly from the funds of the State or the civic municipalities. The primary or more important object of all such institutions is to mitigate bodily suffering, whether that arises from natural or accidental causes, in which respect they are indispensable as a refuge to all who are unable to pay for private medical or surgical aid, or as a convenient means of succour on emergencies to persons of every rank and degree of opulence. While such is the main object of these benevolent institutions, they are in numerous instances serviceable as schools for medicine and surgery; as such, no university, at which these and kindred branches of learning are taught, can be said to be complete without the adjunct of a well-organised hospital, where professors can practically educate their pupils by pointing out varieties of disease and injuries, and exemplifying methods of treatment.

A considerable accession to the number of hospitals took place in the reign of George II., when society became alive to the value of such institutions. It was at this period that the Royal Infirmary of Edinburgh was established (1736). The antiquity of British hospitals sinks into insignificance in comparison with that of some institutions of that kind on the continent. The Hôtel Dieu, in Paris, which is alleged to be the most ancient hospital in Europe, was founded in the seventh century, and long known as the Maison Dieu, received the benefactions of successive sovereigns. It is now conducted on a stupendous scale. Houses of this kind in France usually receive valuable aid from a religious sisterhood, renowned for its practical benevolence, the Sisters of Mercy. A striking example of these women's unselfish and useful labours is furnished at the great hospital for the sick at Lyons, where the entire establishment—cooking, nursing, dispensing medicine, etc.—is gratuitously conducted by them.

No part of the social economy of European countries is so perfect in its organisation, so purely humane, and so unobjectionable on the score of promiscuous charity, as the institution of public hospitals or infirmaries. As means of relief and schools of medicine, they appear to be absolutely essential to every dense community; not the least of their valuable qualities being that, by their prompt and liberal action, they interpose to stem contagious distempers which, if unchecked on their outbreak, might visit and decimate families who are far removed above the need of gratuitous medical attendance.

### ST. BARTHOLOMEW'S HOSPITAL.

St. Bartholomew's Hospital, the earliest institution of the kind in London, was founded A D. 1123 by Rahere, who also founded the priory of St. Bartholomew. The hospital had an independent constitution and a separate estate, but was for some purposes under the control of the priory. It had a master, eight brethren and four sisters, and its community was subject to the rule of St. Austin. From the beginning it was a hospital for the sick, and not a mere almshouse, and this is distinctly expressed in a grant of privileges to it by Edward III., which there states the uses of the hospital; "Ad omnes pauperes infirmos ad idem hospitale confluentes quosque de puerperio surrexerint, necnon ad omnes pueros de eisdem mulieribus genitos, usque septennium, si dictae mulieres intra hospitale praedictum decesserint." The two foundations were finally separated on the dissolution of the priory in 1537. In 1544, at the petition of Sir Richard Gresham, Lord Mayor, and father of Sir Thomas Gresham, Henry VIII. refounded it by Royal Charter, and in 1547 granted it a new charter which gave back to the foundation the greater portion of its former revenues "for the continual relief and help of an hundred sore and diseased," being "moved thereto with great pity for and towards the relief and succour and help of the poor, aged, sick, low, and impotent people . . . lying and going about begging in the common streets of the City of London and the suburbs of the same," and "infected with divers great and horrible sicknesses and diseases." The earliest medical book due to St. Bartholomew's Hospital was written by John Mirfield, one of the canons of the priory in the latter half of the fourteenth century. It is called *Breviarium Bartholomei*, and is a general treatise on medicine, based in part on observations made in the hospital. The immediate superintendence of the hospital was committed at first to Thomas Vicary, Serjeant Surgeon to Henry VIII., Edward VI., Mary and Elizabeth, and author of "The Englishman's Treasury," the first work on anatomy published in the English language. Harvey, the discoverer of the circulation of the blood, was physician to the hospital for 34 years (1609–1643).

"When Henry VIII., in the last year of his reign, founded the hospital anew, he gave 500 marks per annum towards its maintenance, on condition that the city also on their part should give another 500 marks a year, which they agreed to do. But when the city took a survey of what was given by the King for this yearly sum they found the raising of this 500 mark rent to lie only in certain houses, some in great decay, and some rotten and ruinous, and some to whom better tenants had happened, already leased out at terms and rent scarcely sufficient for the help of the poor, so that to make them again work the wonted revenue, and then to continue in the same was no small charge. Pensions also were issuing out of the 500 marks, and granted by letters patent of that King to the hospitaler then, and to other ministers of the same.

"The citizens nevertheless were not discouraged with the evil doings of others, and the great fall of their hopes, but provided with what speed they could to the redress of the decays, disorders, and defaults, and bestowed thereabout not much less than £1,000; whereby in King Edward VI. time it came to such a point that it was fit to receive the number, and to succour them with all necessaries requisite; and accordingly received them, and maintained them. But within five years, after the citizens had the care of this hospital, they were, and that even in pulpits, exclaimed against as if they had wronged this charity, by this mistaken supposition, that this hospital should have made a general sweep of all poor and afflicted, as though the city had abused this charity, and so for their care were rewarded with nothing but open detraction. In this season, notwithstanding, were healed of the pox, fistula, filthy blains and sores, to the number of 800, and thence safe delivered, that others having need might enter in their rooms; besides eight score and twelve that died there in their intolerable miseries, which might have died and stunk in the noses of the city.

"At this first erection the hospital was taken care of by two ranks of persons, viz., governors and officers. There were three surgeons in wages of the hospital, giving daily attendance upon the cures, and a minister, who was the visitor of Newgate according to his office and charge. Now for the charges of St. Bartholomew's, as it was in the reign of King Edward VI. There were certain charges, and uncertain. Under the uncertain come the moneys laid out for shirts, smocks, and other apparel for the poor, for sugar and spices, for candles for the sick, flax for shirts, and weaving of the same, cloth for winding sheets, bowls, brooms, baskets, incense, juniper ashes to buck their clothes. Also money given to them at their departure; which is measured according to their journey and need; which uncertain charges amounted one year to the sum of £60. The certain charges arose from the yearly wages and fees of officers and servants, and the charges of household, reparations, etc. To the hospitaler, £10; to the renter clerk, £10; the butler, £6 13s. 4d.; the cook, £6; the porter, £6; the three surgeons, £60; eight beadles, £26 13s. 4d.; for liveries, £10; the matron and twelve sisters, £26 6s. 8d.; the matron for her board wages at 1s. 6d. per week, £3 18s.; twelve sisters for their board wages at 1s. 4d. per week each, £40 12s.; the matron for her livery, 13s. 4d.; the sisters for their livery, £6; the ministers of Christ's Church by the King's assignment, that is to say, a vicar, a visitor of Newgate, five priests, two clerks, and a sexton yearly, £106; the ministers of the Church within the hospital by the same assignment, that is to say, to a vicar, a clerk, and a sexton, £23 6s. 8d.; certain men of law and other persons, given in fees by the said King's Majesty, yearly by patent, £28 4s.

"For the diets of 100 persons at 2d. per day £300 6s. 8d.; 68 loads of coal at 16s. per load, £54 8s.; for wood yearly, £24; for candles yearly, £5; for yearly reparation of the hospital and tenements appertaining to the same, £40. Sum of the charges certain, £798 2s. Toward which was yearly received, by the King's endowment, £333 6s. 8d.; and by the like endowment of the City of London, £333 6s. 8d." ("Stow's London," vol. 1, p. 184).

The date of the actual commencement of a medical school is unknown, but in 1662 students were in the habit of attending the medical and surgical practice; and in 1667 their studies were assisted by the formation of a library "for the use of the Governors and young university scholars." A building for a museum of anatomical and chirurgical preparations was provided in 1724 and placed under the charge of John Theke, then assistant surgeon to the hospital; and in 1734 leave was granted for any of the surgeons or assistant surgeons "to read lectures in anatomy in the dissecting-room of the hospital." The first surgeon who availed himself of this permission was Mr. Edward Nourse, whose anatomical lectures, delivered for many years in or near the hospital, were followed in 1765 and for many years after by courses of lectures on surgery from his former pupil, Perceval Pott, who held the office of surgeon to the hospital, and numbered among his pupils John Hunter. Further additions to the course of instruction were made by Mr. Abernethy, who was elected assistant-surgeon in 1787, and by whom, with the assistance of Drs. William and David Pitcairn, the principal lectures of the present day were established. Abernethy lectured on anatomy, physiology, and surgery in a theatre erected for him by the Governors in 1791, and his high reputation attracted so great a body of students that it was found necessary in 1822 to erect a new and larger anatomical theatre.

The great quadrangle (200 feet by 160 feet) was designed by James Gibbs, the architect of the Church of St. Martin-in-the-Fields. The first stone was laid June 9th, 1730, and it was completed in 1770. The gate towards Smithfield was built in 1702, the laboratory in 1793 by George Dance, R.A., and the new surgery in 1842. The school of medicine, rebuilt in 1878–9, is a substantial structure of granite and Portland stone, designed by E. I'Anson. One of the greatest individual benefactors to the hospital was the celebrated Dr. Radcliffe, who left the yearly sum of £500 for ever towards improving the diet of the hospital, and the further sum of £100 for ever for the purchase of linen.

